# A Systematic Review of Epidemiology and Risk Factors Associated With Chinese Inflammatory Bowel Disease

**DOI:** 10.3389/fmed.2018.00183

**Published:** 2018-06-19

**Authors:** Guanglin Cui, Aping Yuan

**Affiliations:** ^1^Research Group of Gastrointestinal Diseases, The Second Affiliated Hospital of Zhengzhou University, Zhengzhou, China; ^2^Faculty of Health Science, Nord University, Bodø, Norway

**Keywords:** inflammatory bowel disease, ulcerative colitis, Crohn's disease, incidence, prevalence, China

## Abstract

**Background:** Recent epidemiological data have revealed a dramatically rising prevalence and incidence of inflammatory bowel diseases (IBDs) in Mainland China, a rapidly growing industrialized region, over the last two decades.

**Objectives:** We performed a systematic review to investigate the changing trends in the incidence of IBD in Mainland China and summarized the recent findings in risk factors associated with Chinese IBD.

**Methods:** Relevant references were obtained from an electronic database search via MEDLINE and EMBASE (for English literatures), the China Academic Journals Full-text Database (CJFD) and the China Science Periodical Database (CSPD) in Wanfang Data (for Chinese literatures).

**Results:** Total 1,584 abstracts in Chinese and 171 abstracts in English were collected. Eight full-text with epidemiological data, 25 with risk factor data in Chinese and 7 full-text with epidemiological data, 12 with risk factor data in English were finally identified and included for analysis. Data from included epidemiological studies has showed a striking increase in the incidence rate of IBD in Mainland China over time, and current incidence rates for IBD, UC, and CD were 1.80 (IBD), 1.33 (UC), and 0.46/1,000,000 (CD), though it varies among regions and ethnic minority populations. In addition, several risk factors including environmental factors, diet, intestinal infectious agents, hygiene, stress, and lifestyle have been reported to be associated with the increased incidence of Chinese IBD.

**Conclusion:** This systematic review revealed an increased incidence of IBD in Mainland China. Although it is still lower than that in the Western world, however, China has a huge population; therefore, the total number of IBD patients might not be so little as previously thought and the disease burden of IBD in China is likely underestimated.

**HIGHLIGHTS**

- Recent epidemiological data have revealed a dramatically rising prevalence and incidence of inflammatory bowel diseases (IBD) in Mainland China, a rapidly growing industrialized region, over the last two decades.
- This systematic review based on recent epidemiological data has revealed a striking increase in the incidence rate of IBD in Mainland China, though it varies among regions and ethnic minority populations.
- Several potential risk factors of IBD including environmental factors, diet, intestinal infectious agents, hygiene, stress, and lifestyle have been reported to be associated with the increased incidence of Chinese IBD.
- This systematic review on epidemiologic and risk factor studies has expanded understanding of the occurrence, distribution, geographic variance and risk factors of Chinese IBD and will provide clinicians important information in understanding current status of IBD in Mainland China.

## Introduction

Inflammatory bowel diseases (IBD) are a group of chronic intestinal inflammatory diseases that mainly include ulcerative colitis (UC) and Crohn's disease (CD) (1). Although the etiology of IBD remains unclear, it is widely considered that environmental factors, genetic predisposition, and dysregulated immune response may strongly increase the risk for the development of IBD (2). Pathologically, the inflammation in patients with UC is limited to the colonic and/or rectal mucosa, while in patients with CD, it may affect any part of the digestive tract. IBD includes a variety of symptoms such as abdominal pain, diarrhea, stool with blood and mucus, episodes of remission and relapse and a generally poor quality of life. The treatment for IBD relies on medicines including five aminosalicylic acids (5-ASAs), glucocorticoids, immunomodulators and immunotherapeutic agents. However, drug resistance and disease relapse occur very often, which makes the treatment for some patients difficult.

The timing of the expansion of IBD varies between Western and Eastern countries. IBD was initially identified in Western countries during the industrial revolution but was rarely reported in Asia, including China, for a long period. Therefore, IBD was traditionally considered a common intestinal inflammatory disorder of Western countries. China's first case of UC was described in 1936, and the first case of CD in approximately 1950 (3). The incidence and prevalence of IBD remained low for a long period. Therefore, gastroenterologists were not familiar with IBD, and their diagnostic pathway was one of exclusion. In addition, many medical students could only read about IBD in textbooks and Western literature but had no much chance to observe it in patients. Before the 1970s, only case reports were available, and they were predominately on the clinical diagnosis and management. Basic studies on the etiology, pathogenesis, risk factors, genetics and immunology were often ignored or unavailable.

However, the geographic spread of IBD has rapidly changed over the last two decades (4–7). A striking increase in IBD in Asia has been observed (4, 8). Similarly, recent hospital-based reports from Mainland China have noted an increasing incidence of IBD (9, 10). IBD has become a common and important health problem in China and is a current challenge for patients, clinicians and health administrators. However, today, most Chinese health administration districts do not conduct the registration of IBD patients, one of reasons for the lack of nation-wide population-based epidemiological data. In addition, most existing reports have been written and published in Chinese, which limits the spread of information to other non-Chinese-speaking populations. We have therefore systematically reviewed the recent literature, summarized the current progression of clinical epidemiology and risk factors associated with IBD that predominantly occurs in Mainland China.

## Methods

### Literature search

**Figure 1:**
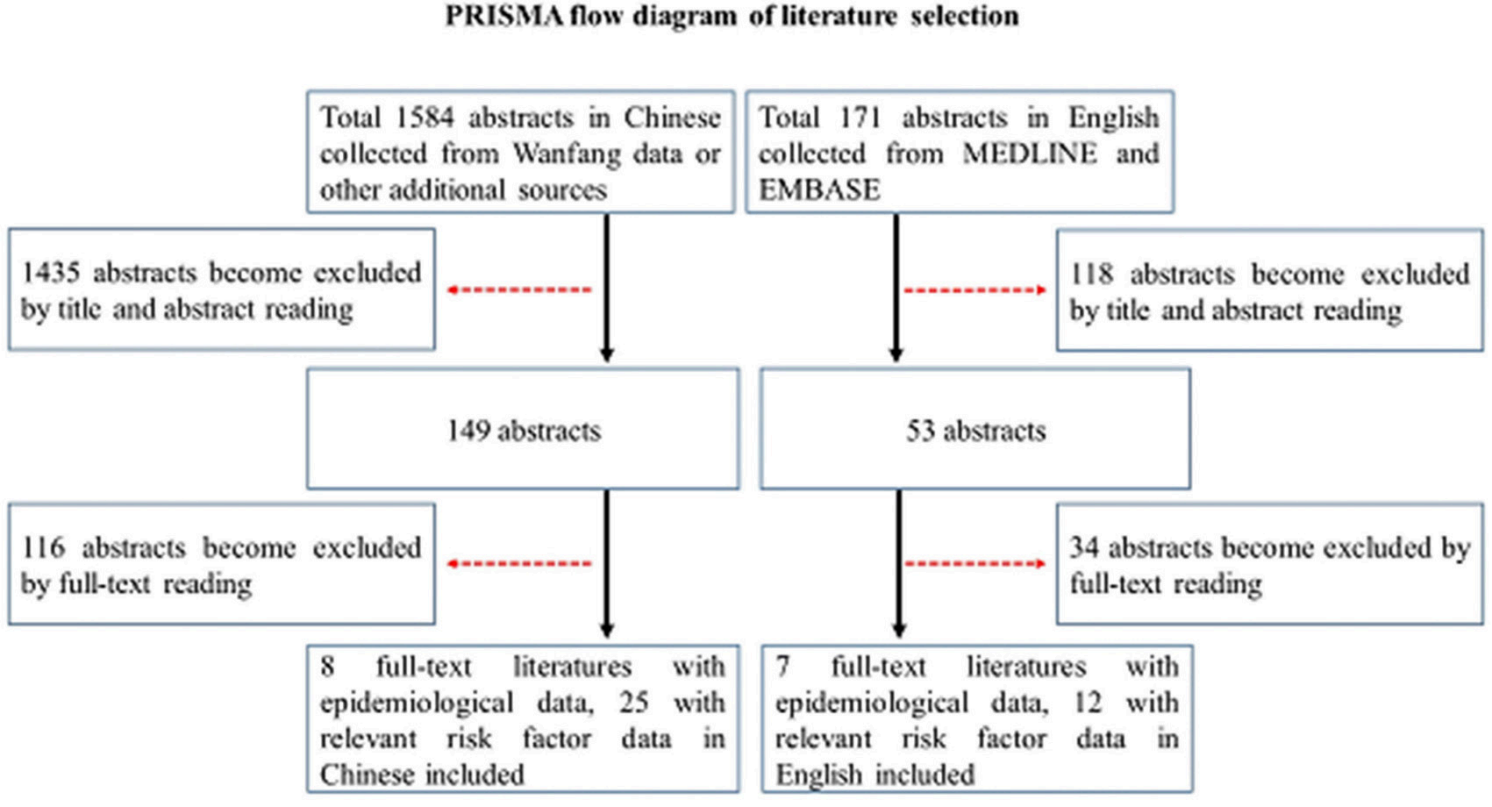
PRISMA flow diagram of literature selection.

For this review, relevant references published in English were obtained from an electronic database search via MEDLINE and EMBASE by the authors using the search terms “inflammatory bowel diseases,” “ulcerative colitis,” “Crohn's disease,” and “Chinese.” Additionally, relevant references published in Chinese were obtained from an electronic database search via China Academic Journals Full-text Database (CJFD) in the China Knowledge Resource Integrated Database, China Science Periodical Database (CSPD) in the Wanfang database by the authors using the same search terms in Chinese following PRISMA methodology (Figure 1). The search period comprised between 1980 and 31st December 2017. After screening the abstracts, the articles deemed relevant were cross-referenced for additional manuscripts.

### Eligibility criteria

We used the following eligibility criteria to include papers in this systematic review: (1) article written in English or Chinese; (2) original data from Mainland China; (3) only studies performed in humans; (4) individual patient information; (5) full-text available; (6) studies that encompass both pediatric and adult patients diagnosed with IBD.

### Data collection and extraction

Data collection included the first author's name, year of publication, language of publication, study location, number of subjects, mean age of subjects, proportion of male subjects.

Two independent reviewers extracted the data of included studies and discrepancies in data interpretation were resolved by consensus. The quality of the incidence and prevalence studies were assessed by whether the clinical diagnostic criteria were clearly defined and used.

### Synthesis of the evidence

We extracted the same information from the included studies. A meta-analysis of all included studies was not be conducted because of study population heterogeneity and the relevant differences on the methodology of the included studies.

## Results

### Description of studies

The searches yield a total of 1,584 abstracts in Chinese collected from Wanfang data or other additional sources and 171 abstracts in English collected from MEDLINE and EMBASE. After reading titles and abstracts, duplicates, non-English or Chinese articles, animal or immunologic studies, studies not involving Chinese UC, CD, or IBD were excluded. Total 1,584 abstracts in Chinese collected from Wanfang data or other additional sources for further evaluation. Eight full-text with epidemiological data, 35 with risk factor data literatures in Chinese and 7 full-text with epidemiological data, 12 with risk factor data literatures in English were finally included for analysis (Figure 1).

### Incidence and prevalence of IBD in Mainland China

#### IBD incidence and prevalence in different districts of Mainland China

We first analyzed current IBD incidence rate of IBD, UC, and CD in mainland China, average incidence rates were 1.80 (IBD), 1.33 (UC), and 0.46/1,000,000 (CD) during the period of 2010–2013 based on included epidemiological data.

**Figure 2:**
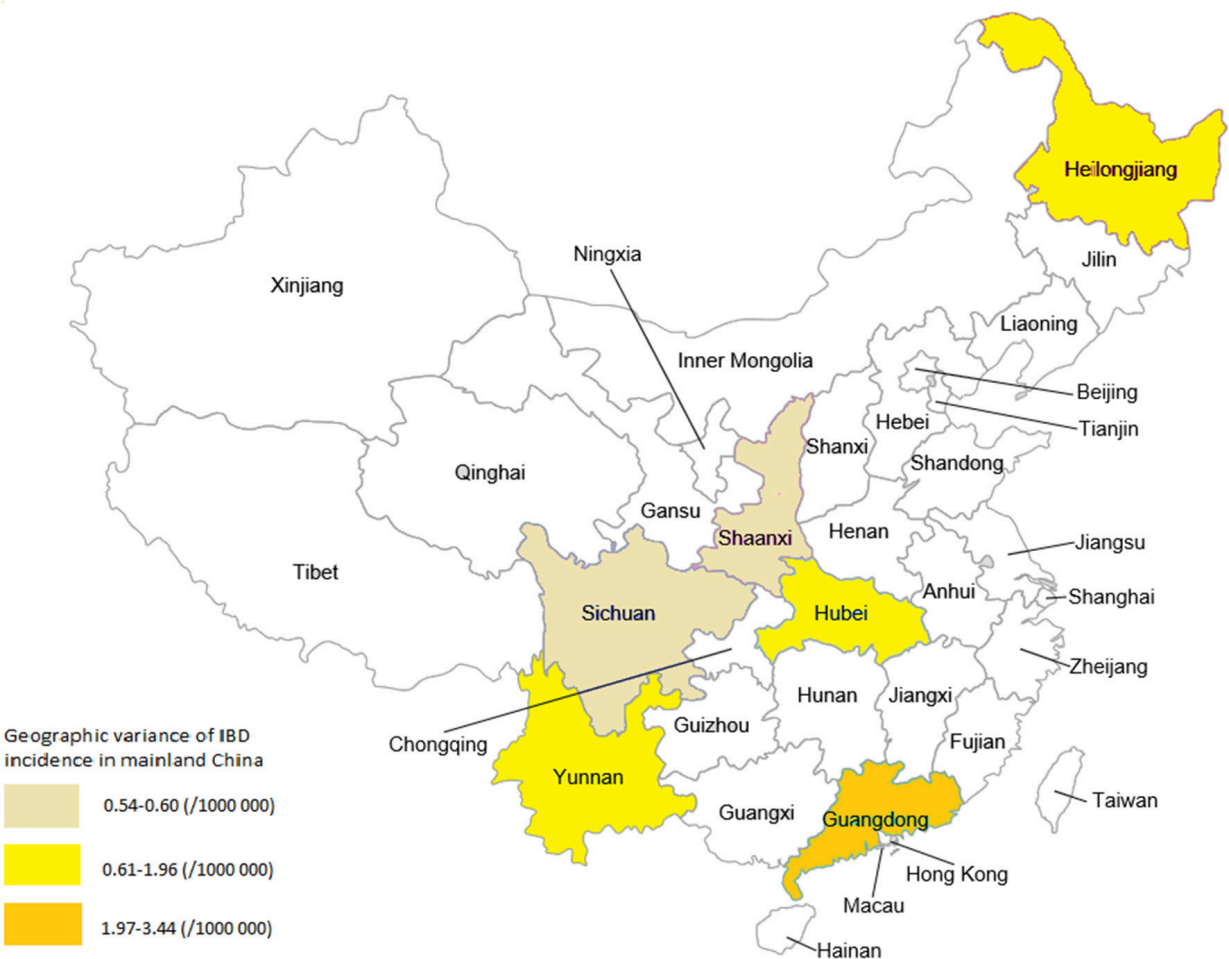
Geographic variance of IBD incidence in different geographic regions in Mainland China.

Variations of IBD incidence and prevalence between different districts of Mainland China was examined. Among included epidemiological studies, the lowest incidence of IBD was reported in Sichuan (Chengdu city, located in South-western China) and Shanxi (Xian city, located in Western China) provinces, at a rate of 0.54~0.60/1,000,000; the highest incidence rate was in Guangdong province (Southern China) at a rate of 1.97~3.44 (see Figure 2). Whereas the incidence rate of IBD in Yunnan (South-western China), Hubei (central China) and Heilongjiang (Northern China) was between 0.61 and 1.96/1,000,000. In detail, incidence rates of UC and CD at 2.05/100,000 and 1.09/100,000 person-years in Guangdong province (Southern China) (11), 1.64/100,000 (UC) and 0.13/100,000 (CD) person-years in Daqing City, Heilongjiang Province (Northern China) (10), and 1.45/100,000 (UC) and 0.51/100,000 (CD) person-years in the central Chinese region of Wuhan (12). Ng et al. have reported a very similar annual IBD incidence of 0.58/100 000 (UC: 0.43 and CD 0.14/100,000, respectively) in Chengdu (a city located in South-western China), and 0.54/100,000 for IBD (UC: 0.42 and CD 0.07/100,000, respectively) in Xian (Western China) (5). From those data, we find that both the UC and CD incidences in Mainland China appear to be higher in Southern China (regions with the greatest urbanization and most economic development) than in the Northern region (13) (see the summary in Table 1).

**Table 1:** Incidence and prevalence (per 1,000,000) of IBD in different geographic regions in Mainland China.

**Region**	**Study period**	**Crude incidence**			**Crude prevalence**		
		**IBD**	**CD**	**UC**	**IBD**	**CD**	**UC**
Mainland China (14)	1950–2000		0.28			1.38	
Zhongshan (Guangdong Province, Southern China) (11)	2011–2012	3.14	1.09	2.05	–	–	–
Guangzhou (Guangdong province, Southern China) (7)	2011–2012	3.44	1.22	2.22	–	–	–
Wuhan (Hubei province, Central China) (9)	2010–2011	1.96	0.51	1.45	–	–	–
Xian (Shanxi province, North western China) (7)	2011–2012	0.54	0.07	0.42	–	–	–
Chengdu (Sichuan Province, Southwestern China) (7)	2011–2012	0.58	0.14	0.43	–	–	–
Yunnan (Yunnan Province Southwestern China) (15)	1998–2013	0.07 (1998) 1.15 (2013)	0 (1998) 0.08 (2013)	0.07 (1998) 1.08 (2013)	0.25 (1998) 7.45 (2013)	0.24 (1998) 7.04 (2013)	0.005 (1998) 0.42 (2013)
Daqing (Heilongjiang Province, Northern China) (10)	2012–2013	1.77	0.13	1.64	–	–	–

*Geographic region (reference numbers)*.

**Figure 3:**
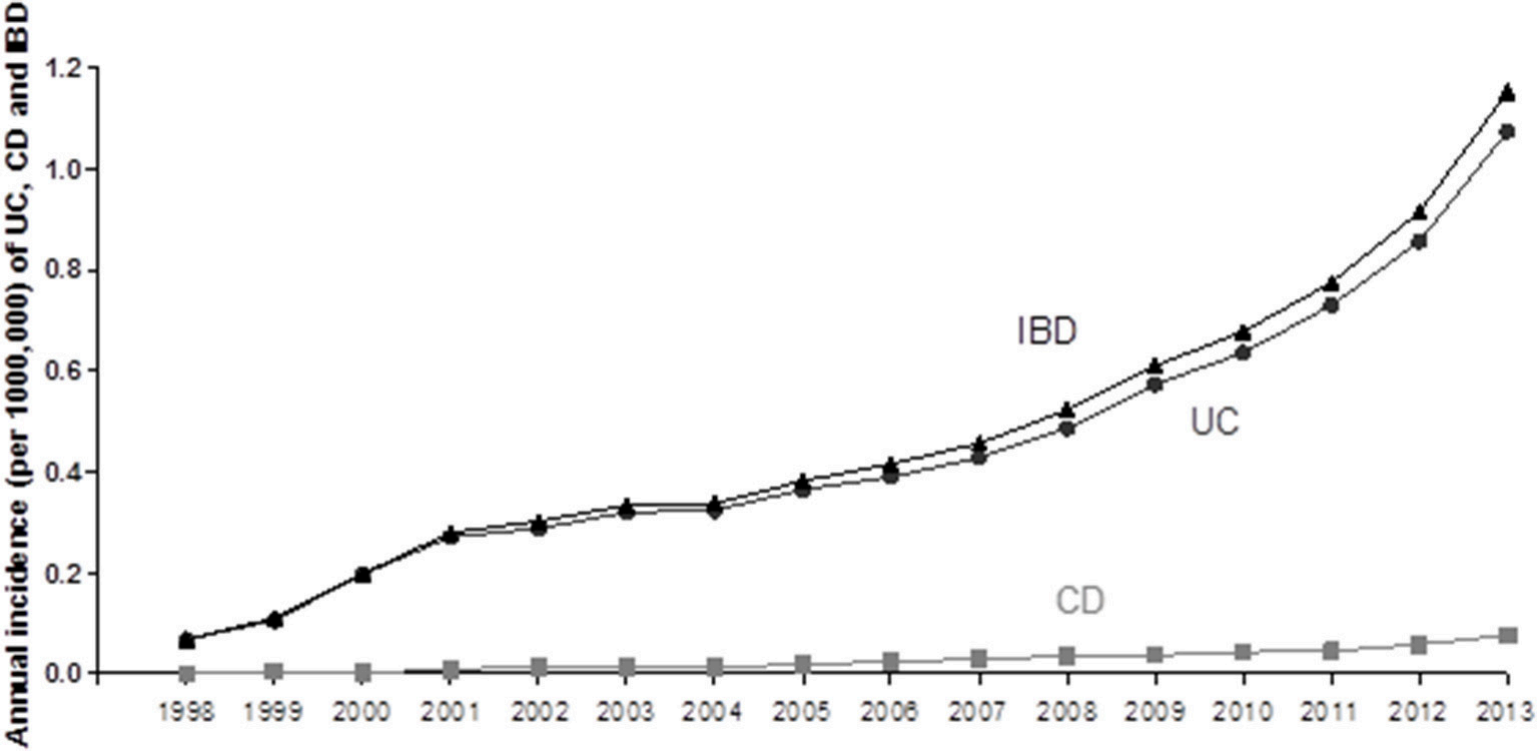
The incidence changing trend of UC, CD, and IBD from 1998 to 2013 in Yunnan province, China.

Time changing trend of IBD incidence rate in Mainland was further examined. One of included studies has investigated the changing trends of the incidence and prevalence of IBD between 1998 and 2013 (total 16 years) in Yunnan province (located in South-western China) (15). A striking increase trend in the incidence rate of IBD over time was illustrated (see Figure 3). They found that both the prevalence and incidence in this province showed a significant increasing trend in this province (15). The prevalence of IBD, UC and CD per 100,000 people has remarkably risen from 0.246 (for IBD), 0.241 (for UC) and 0.005 (for CD) in 1998 to 7.453 (for IBD), 7.035 (for UC) and 0.418/100,000 (for CD) in 2013. In the meantime, the incidence of IBD in this province also increased from 0.068 (for IBD), 0.068 (for UC), and 0/100,000 (for CD) in 1998 to 1.152 (For IBD), 1.075 (for UC), and 0.077/100,000 (for CD) in 2013 (Figure 2). Compared to other developed provinces in Mainland China, the prevalence and incidence of IBD, UC and CD in Yunnan province are lower. Another included study analyzing 3,618 Chinese CD patients on the Mainland has also suggested an uneven distribution of IBD cases; patients were predominately in the northern, eastern, and southern regions of China (16). This could be due to the rapidly expanding urbanization and developing industry occurring in the above regions.

#### IBD in different Chinese ethic populations

China is a country with 56 ethnic minorities; many ethnic minorities live in regions with different geographic features, economic development levels, genetic predispositions, environments, and lifestyle habits. Thus, it is important to analyze changed IBD incidence and prevalence in different Chinese ethnic populations.

Yunnan Province, located in South-western China, has 51 ethnic minorities, and populations of ethnic minorities account for 33.41% of its total population. Among the ethnic minorities, 26 have a population of more than 5,000 within a fixed living region. The epidemiological data from this province showed that among 18 different ethnic minorities, the highest prevalence of total IBD was observed in the Han population (9.463/100,000), followed by the Hui (Muslim) minority population (8.563/100,000), Man minority population (7.092/100,000) and Bai minority population (5.271/100,000). The highest prevalence of UC was also found in the Han nationality (8.918/100,000), followed by the Hui population (7.720/100,000), Man population (7.092/100,000), and Bai population (5.083/100,000). The highest prevalence of CD was recorded in the Tibetan ethnic minority group population (1.376/100,000), followed by the Hui minority group (0.842/100,000 and Han: 0.545/100,000) (15, 17).

#### IBD in Chinese children

Approximately 25% of IBD patients were diagnosed under the age of 18 years, presenting with more extensive distribution and severity of disease than adult onset. Therefore, we have analyzed the pediatric IBD in Mainland China. We found only one published study that examined the increasing trend in pediatric IBD incidence in Shanghai (the biggest city located in eastern China) from 2000 to 2010 (18). The year-specific incidence rate of IBD in 0–14-year-old children is rising steadily, from 0 in 2000 to 6.051/100,000 in 2010 (18). The authors also analyzed the clinical characteristics of pediatric IBD in Shanghai (18). They found that most pediatric patients with IBD had a mild or moderately active disease (18).

### Potential risk factors associated with Chinese IBD

Several risk factors for Chinese IBD have been postulated in included studies from mainland China (19–22), and we have summarized these in Table 2.

**Table 2:** Current potential risk and protective factors for Chinese IBD patients.

**Factors**	**CD**		**UC**	
	**Risk**	**Protective**	**Risk**	**Protective**
Family history			+(23)	
Infectious bowel disorders			+(24)	
Smoking	+(25)			±(26, 27)
Specie food			+(28)	
Tap water consumption				+(28)
Heavy sugar consumption	+(21)		+(28)	
Drinking tea				+(26, 27, 29)
Breastfeeding				+(26, 27)
Often feeling stress			+(28)	
Crowded living conditions	+(25)			
Use of toothpaste	+(25)			
Daily consumption of eggs	+(25)			
Frequent gastrointestinal and respiratory infection during childhood	+(25)			
Bean consumption		+(30)		
Regular physical activity		+(15, 31)		
Inconsistent dining hours	+(15, 31)		+(15, 31)	
Often eating fried food			+(15)	
History of allergy			+(15)	
Using antibiotics frequently before the age of 14 years			+(15)	
Appendectomy	+(15)			

*+(reference numbers)*.

#### Family history

Family history could be a risk factor for Chinese IBD (23, 24). Yuan et al. analyzed the risk factors in a cohort of 196 Chinese patients with UC and found that only 5.6% of patients had a positive family history, which is lower than the reported rate of 10~20% in Western countries (23).

#### Smoking

Unlike the reports from western counties, whether smoking is a protective factor or a risk factor for IBD in the Chinese population is still unsettled (26, 27).

#### History of infectious bowel disorders in the past

Surveys performed in Chinese IBD patients reported that infectious diarrhea in the past may be a risk factor (24–27). Miao et al. reported that a history of allergies, intestinal infectious diseases and using antibiotics frequently before the age of 14 years may increase the risk for UC later (15, 31).

#### Diet

Several analyses suggested that heavy fried food intake, spicy food intake, too much sugar intake, daily consumption of eggs and milk intake might be risk factors (25–28), whereas drinking tap water was a protective factor for UC (28). Families with refrigerator showed a lower risk for CD (25). Another study analyzed the risk of consuming different foods in 41 Chinese cases of CD and found that bean consumption was a protective factor, potentially due to the high content of omega-3 (30). A case-control study performed in multiple Chinese medical centers confirmed that heavy consumption of sugary foods and meats could increase the incidence of CD (21). Miao et al. performed a nested case-control study in Yunnan province to examine environmental risk factors in patients with IBD (15, 31). They revealed that an inconsistent dining time might be a risk factor in both UC and CD (15, 31). They found that peoples with dining not on time >3 times/week was associated with increased risk of UC (Odds ratio 2.087, 95%CI 1.394~3.127) and of CD (Odds ratio 1.876, 95%CI 1.807~3.236) (15).

#### Genetic predisposition

In recent years, the important role of genetic background in the development of CD has been hypothesized (32). However, current evidence from Chinese CD patients suggests that individual genetic background may have a slight effect on the pathogenesis of IBD in China (7, 33, 34). For example, studies from Western nations have suggested a close relationship between NOD2 (nucleotide-binding oligomerization domain 2) /CARD15 gene polymorphisms and CD. Several studies have assessed such relationship in Zhuang patients with CD (one of Chinese ethic populations in Guangxi Province) (35) and Han patients with CD (36, 37). However, these studies found that the common variants in NOD2/CARD15 found in Caucasians with CD are not associated with CD in the both Chinese Zhuang and Han population. Further analysis also revealed that CARD15 gene polymorphism was not associated UC in Chinese populations (38, 39). Studies comparing IBD-related susceptibility genes between Chinese patients and Western patients might answer this question in the future.

#### Others

There are studies showing that regular physical activity is a protective factor, while stress is a risk factor (15, 31). In addition, drinking tea may be a protective factor for UC, and a history of appendectomy may be an independent risk factor for CD (15, 31).

## Discussion

To the best of our knowledge, high-quality, national-wide, population-based studies of the prevalence, and incidence of IBD that cover Mainland China are currently unavailable. When summarizing the existing epidemiological data in this systematic review, it demonstrated that the incidence and prevalence of IBD in different districts of Mainland China have shown an overall upward trend (3, 40, 41), though they are still lower than the reported rates in European countries and the United States (2).

Recently, Li et al. have performed a meta-analysis to summarize the incidence of Chinese IBD (42), and they found that the incidence rate for all IBDs is 1.74/100,000, while for CD and UC, the rates are 0.40/100,000 and 1.18/100,000, respectively, based on the recently published epidemiological data for UC and CD in different Chinese regions. Our current analysis revealed that the incidence rates in Chinese populations were 1.33 (UC) and 0.46/1,000,000 (CD) during the period of 2010–2013 based on included epidemiological studies and comparable to the overall incidence rates of CD and UC between 2000 and 2010 in Taiwan, which were 0.208 and 0.838/100 000 (43). These data are also similar to the incidence of UC and CD (1.95 and 0.51/100,000, respectively) reported during the year 1991 in Japan (44) and Korea populations (1.74 and 0.52/100,000, respectively) between 1986 to 2005 (45). The incidence rate of UC and CD in industrialized regions of China for example Guangdong province are 1.09 for CD, 2.05/1,000,000 for UC during the period of 2011–2012 (11); which are still lower than that in Korean population (4.6 and 3.2/100,000) during the period of 2006–2012 (46).

A geographic influence is evident in different ethnic populations (47). China is a large country with the biggest population in the world, and geographical differences are quite large between regions. Indeed, regional variations in IBD prevalence and incidence in different districts across Mainland China have been reported (16). Previously, some analyses have revealed the variance in incidence rates between different geographic regions including Chinese populations in both Hong Kong and Taiwan (42). From current analysis, we were able to confirm such geographic variance and find that both the UC and CD incidences in Mainland China appear to be higher in Southern China (regions with the greatest urbanization and most economic development) than in the Northern region (13) (see the summary in Table 1). This could be due to the rapidly expanding urbanization and developing industry occurring in Southern China regions.

In general, although the incidence and prevalence are still lower than those in most Western countries [see review (48)], an overall upward trend is emerging (14, 49, 50), particularly considering the fact that China is a developing country with the biggest population in the world. The total number of IBD patients in China might not be much less than that in the Western world.

The variation in the incidence and prevalence of IBD between different ethnic populations has been previously demonstrated in Western nations (2). China is a country with 56 ethnic minorities; many ethnic minorities live in regions with different geographic features, economic development levels, genetic predispositions, environments, and lifestyle habits. Epidemiological data from Yunnan Province showed that among 18 different ethnic minorities, the highest rate of both prevalence and prevalence of total IBD and UC were found in Han population. However, CD is more frequently seen in the Tibetan ethnic minority group population. This study provides first time the difference of IBD incidence and prevalence in different Chinese ethic populations. However, this information is preliminary and limited by the small sample size and geographic regions. Further studies are still needed.

Since 25% of IBD patients were diagnosed under the age of 18 years, the incidence, prevalence and clinical outcomes of pediatric IBD have emerged as the key clinical issues (51, 52). Results from different Western counties have shown that in children with IBD, especially children with CD, the incidence has significantly increased over time (53). Because there are no large population-based epidemiological data available in Chinese children, the true disease burden of IBD among them remains unclear. One included study in this review has examined the pediatric IBD incidence in Shanghai from 2000 to 2010 has revealed an increasing trend in this biggest city located in eastern China over time (18). However, Shanghai is a well-developed industrial city and may have a higher incidence of pediatric IBD than other regions of Mainland China; more studies performed in other regions still needed.

Epidemiologic studies from different Chinese regions have investigated the potential risk factors for Chinese IBD (19–22), in which some factors are similar to that reported in Western IBD patients and some factors have not been confirmed in Chinese IBD patients. For instance, only 5.6% of Chinese UC patients had a positive family history, which is lower than the reported rate of 10~20% in Western countries (23). Moreover, whether smoking is a protective factor or a risk factor for IBD in the Chinese population is still unsettled (26, 27). In Chinese patients, it has been reported that a history of allergies, intestinal infectious diseases and using antibiotics frequently before the age of 14 years may increase the risk for UC later (15, 31). In addition, diet taking not on time >3 times/week and physical activity have been found to be the risk factors of UC and CD for Chinese populations, which might partial explant the increase incidence in some regions of China (15). Regarding the role of genetic predisposition, current Chinese studies suggests that individual genetic background has a slight effect on the pathogenesis of IBD in China (7, 33, 34). Strong epidemiological evidence have supported the view that increased IBD burden among Asians could be a result of environmental factors i.e. the westernization of lifestyle and changing diet habits, and possibly also relating to fast industrialization (54). There are some studies to investigate the incidence rate of IBD in migrants from low incidence areas for example Asia to high incidence countries (Western nations), and found that first-generation immigrants had a higher chance of developing IBD than people in original countries (55, 56). These findings may remain us that epidemiological comparison of IBD incidence between Chinese immigrants living in Western countries and Mainland China should be considered in the future.

In conclusion, growing evidences show that IBD is dramatically increasing in Mainland China, although the incidence and prevalence of IBD remain lower than those in developed countries. In addition, several risk factors including environmental factors, diet, intestinal infectious agents, hygiene, stress, and lifestyle have been reported to be associated with the increased incidence of Chinese IBD. As a big county with a large population and ongoing rapidly expanding urbanization and westernization and changing environmental factors, China may have a much higher number of IBD cases than previously thought. The true disease burden of IBD in Mainland China should not be underestimated. It is time for us to sound the alarm and the priority for the future is to register patients with IBD in different geographic locations and undertake a nation-wide population-based epidemiological study that covers most areas of Mainland China.

## Author contributions

All authors listed have made a substantial, direct and intellectual contribution to the work, and approved it for publication.

### Conflict of interest statement

The authors declare that the research was conducted in the absence of any commercial or financial relationships that could be construed as a potential conflict of interest.

## Abbreviations

IBD: inflammatory bowel diseases
UC: ulcerative colitis
CD: Crohn's disease
NOD2: nucleotide-binding oligomerization domain-2.
